# Analysis of changes in circular RNA expression and construction of ceRNA networks in human dilated cardiomyopathy

**DOI:** 10.1111/jcmm.16251

**Published:** 2021-01-22

**Authors:** Zhenhao Lin, Yongchao Zhao, Fangjie Dai, Enyong Su, Fuhai Li, Yan Yan

**Affiliations:** ^1^ Department of Cardiology Zhongshan Hospital Fudan University Shanghai China; ^2^ Shanghai Institute of Cardiovascular Disease Shanghai China

**Keywords:** circular RNA, competitive endogenous RNA, dilated cardiomyopathy, mRNA, RNA sequencing

## Abstract

Dilated cardiomyopathy (DCM) is a severe life‐threatening disease worldwide, and the underlying mechanisms remain unclear. Circular RNAs (circRNAs) have been reported to play important roles in various cardiovascular diseases and can function as competitive endogenous RNAs (ceRNAs). However, their role in human DCM has not been fully elucidated. In the present study, heart samples from DCM patients and healthy controls were used to identify circRNAs by RNA sequencing. Real‐time quantitative reverse transcription‐polymerase chain reaction (qRT‐PCR) was conducted to validate differentially expressed circRNAs and mRNAs. A total of 9585 circRNAs and 22050 mRNAs were detected in the two groups. Overall, 213 circRNAs and 617 mRNAs were significantly up‐regulated in the DCM group compared with the control group. Similarly, 85 circRNAs and 1125 mRNAs were significantly down‐regulated. According to the ceRNA theory, circRNAs can indirectly interact with mRNAs by directly binding to microRNAs (miRNAs), and circRNAs and mRNAs should be concurrently either up‐regulated or down‐regulated. Based on this theory, we constructed two circRNA‐miRNA‐mRNA networks by using the RNA sequencing data and prediction by proprietary software. Gene Ontology (GO) and Kyoto Encyclopedia of Genes and Genomes (KEGG) pathway analyses were performed to probe the potential functions of differentially expressed circRNAs. In conclusion, this study revealed that the expression of cardiac circRNAs was altered in human DCM and explored the potential functions of circRNAs by constructing ceRNA networks. These findings provide a foundation for future studies of circRNAs in DCM.

## 1 INTRODUCTION

Dilated cardiomyopathy (DCM), characterized by ventricular dilation and systolic dysfunction, is a common disease that results in arrhythmia, heart failure and sudden cardiac death. Many factors have been reported to be closely related to DCM, including coronary artery disease, endocrine and metabolic abnormalities, viral infections, autoimmunity and gene mutations.^1^ However, the exact mechanisms involved in the development of DCM have not yet been elucidated. At present, there is no effective treatment for DCM, and the mortality rate is increasing year by year. Therefore, studies focused on finding novel therapeutic targets for DCM are urgently required.

With the rapid development of sequencing technologies, an increasing number of non‐coding RNAs (ncRNAs), such as microRNA (miRNA), long non‐coding RNA (lncRNA) and circular RNA (circRNA), have been found. Although messenger RNAs (mRNAs) have been studied extensively, traditional protein‐coding RNAs account for only a minority of all RNAs, and ncRNAs actually account for most of the transcriptome.^2^ In the past, ncRNAs were considered evolutionary junk, but increasing studies have indicated that these non‐coding transcripts play important roles via epigenetic, post‐transcriptional and translational mechanisms and have considerable impacts on biological processes.^3^, ^4^, ^5^ Therefore, ncRNAs have been assessed as potential diagnostic candidates and therapeutic targets for various diseases, including cardiovascular diseases.^6^, ^7^

CircRNAs, unlike linear RNAs, which contain 5′ and 3′ ends, have covalently linked ends that form a closed continuous loop. Due to their circular structure, circRNAs are resistant to RNase R activity and are more stable than other RNAs.^8^ Currently, circRNAs can be categorized as exonic circRNAs (ecircRNAs), circular intronic RNAs (ciRNAs) and exon‐intron circRNAs (EIciRNAs) based on the back‐splicing mechanism and the rearrangement of exons and/or introns in precursor messenger RNA (pre‐mRNA). In addition, circRNAs can also be classified into five types, ‘exonic’, ‘intronic’, ‘intergenic’, ‘antisense’ and ‘sense overlapping’ according to their location relationship with adjacent coding RNA.^9^, ^10^, ^11^ Emerging evidence has shown that the vast majority of circRNAs are derived from exons and are primarily localized in the cytoplasm, while only a small portion of circRNAs, particularly ciRNAs, reside in the cell nucleus.^9^, ^12^ Based on their subcellular localization, circRNAs play important roles in regulating gene expression at the transcriptional, post‐transcriptional, translational and post‐translational levels.^13^, ^14^, ^15^ Recent studies have revealed many functions of circRNAs, among which their function as competing endogenous RNAs (ceRNAs) has become a research hotspot. As ceRNAs, circRNAs contain shared miRNA response elements and can competitively bind to miRNAs, resulting in a reduction of miRNAs and an up‐regulation of the expression of miRNA target genes. As circRNAs can ‘absorb’ miRNAs like a sponge, they are often referred to as miRNA sponges.^16^ Studies have revealed that circRNAs are abundant in human tissues, including the heart, and most are tissue‐specific.^17^, ^18^ Interestingly, a number of circRNAs are generated from genes, such as TTN and RYR2, which are associated with cardiovascular diseases.^19^, ^20^ In addition, circRNAs are differentially expressed between healthy and diseased human hearts and peripheral blood, suggesting that they may play important roles in cardiac physiology and the initiation and development of cardiovascular diseases.^21^, ^22^

Recently, studies have identified the microarray profile of lncRNAs and miRNAs in human DCM and constructed a lncRNA‐miRNA‐mRNA network.^23^, ^24^ As the changes in circRNA expression and the potential circRNA‐miRNA‐mRNA network remain unclear in human DCM, the present study evaluated circRNA expression in heart samples from DCM patients and healthy controls and constructed two ceRNA networks based on the ceRNA theory. The results provide a new understanding of the mechanisms involved in the development of DCM. Furthermore, the circRNA‐miRNA‐mRNA network indicated that circRNAs may become potential therapeutic targets for DCM.

## 2 MATERIALS AND METHODS

### 2.1 Samples and RNA isolation

DCM heart samples were collected from the left ventricular wall of explanted hearts of patients diagnosed with DCM (clinical data are presented in Appendix S1). Control heart samples were collected from healthy donors (accident victims). Total RNA was isolated using TRIzol and purified with the RNeasy mini kit (Qiagen) according to the manufacturer's instructions. RNA quantity and quality were measured by a NanoDrop ND‐1000 instrument (Thermo Fisher Scientific). RNA integrity was determined by gel electrophoresis (Appendix S2). The study protocol was approved by the Medical Ethics Committee of Zhongshan Hospital of Fudan University, and informed consent forms were signed by the subjects recruited in the study or by their immediate family members.

### 2.2 RNA high‐throughput sequencing

The removal of rRNAs from total RNA was performed using the NEBNext rRNA Depletion Kit (New England Biolabs, Inc) following the manufacturer's instructions. RNA libraries were constructed by using rRNA‐depleted RNAs with the TruSeq Stranded Total RNA Library Prep Kit (Illumina) according to the manufacturer's instructions. Libraries were quality controlled and quantified using the BioAnalyzer 2100 system (Agilent Technologies). RNA libraries were denatured to single‐stranded DNA molecules, captured on Illumina flow cells, amplified in situ as clusters and sequenced for 150 cycles on the HiSeq 4000 system (Illumina) following the manufacturer's instructions.

### 2.3 CircRNA and mRNA sequencing analysis

Paired‐end reads were obtained from the HiSeq 4000 system and were quality controlled by Q30. Next, 3′ adaptor trimming and low‐quality reads removal were performed using Cutadapt software (V1.9.3). The high‐quality trimmed reads were used to analyse circRNAs and mRNAs. The high‐quality clean reads of circRNAs were mapped to the reference genome using STAR software (v2.5.1b). CircRNAs were identified using DCC software (v0.4.4) and annotated from the circBase database.^25^ The high‐quality reads of mRNAs were aligned to the human reference genome (UCSC hg 19) with hisat2 software (v2.0.4).

### 2.4 Identification of differentially expressed circRNAs and mRNAs

Edger software (v3.16.5) was used to normalize the data and perform differentially expressed circRNA analysis. Cuffdiff software (v2.2.1, part of Cufflinks) was used to obtain the fragments per kilobase of exon per million (FPKM) for the expression profiles of mRNAs, and fold change and *P* value were calculated based on the FPKM values. CircRNAs and mRNAs that exhibited fold change ≥2 or ≤0.5 with *P* value < .05 were considered significantly differentially expressed.

### 2.5 Experimental validation of circRNAs and mRNAs

Quantitative real‐time PCR (qRT‐PCR) was used to validate circRNA and mRNA expression. Three up‐regulated and three down‐regulated circRNAs and mRNAs were selected for validation. For the validation of circRNAs, we performed a divergent PCR protocol by using divergent PCR primers. The divergent primers were designed to span the circRNA backsplice junction sequence, so that they can only amplify the circRNAs, and not the linear RNAs with the same sequence. All the primers used are presented in Appendix S3. Total RNA was reverse transcribed into complementary DNA (cDNA) using the PrimeScript RT Reagent Kit (Perfect Real Time; TaKaRa) following the manufacturer's instructions. The cDNA was subjected to qRT‐PCR analysis on an Applied Biosystems 7500 Fast Real‐Time PCR system. The relative expression was calculated using the 2^−ΔΔCT^ method.

### 2.6 Functional enrichment analysis

Gene Ontology (GO) and Kyoto Encyclopedia of Genes and Genomes (KEGG) pathway analyses were performed of target mRNAs of differentially expressed circRNA. GO analysis included terms in the biological process, molecular function and cellular component categories. The enriched GO terms and KEGG pathways between the two groups are presented as differentially expressed. A *P* value of <.05 was considered to indicate significant enrichment.

### 2.7 Construction of ceRNA networks

CircRNA‐miRNA interactions were predicted using miRNA target gene prediction software. The identification of miRNA‐binding sites and target mRNA prediction were performed using proprietary software based on miRanda and TargetScan. The circRNA‐miRNA‐mRNA network was constructed based on the ggalluvial package in R.

### 2.8 Statistical analysis

The data are expressed as the mean ± SEM and analysed with SPSS 21.0 (SPSS Inc). Student's *t* test was used to determine the statistical significance. *P* values < .05 were considered statistically significant.

## 3 RESULTS

### 3.1 Distribution profiles of circRNAs

**FIGURE 1:**
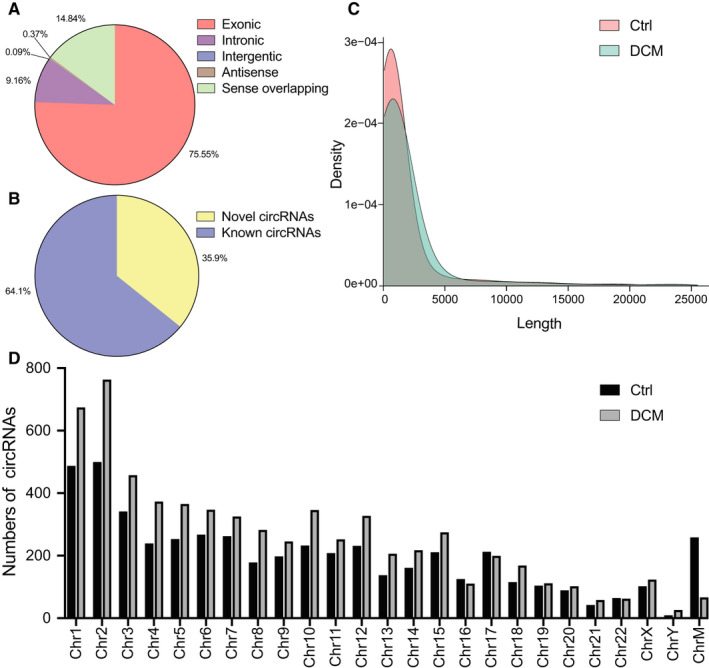
Distribution profiles of circRNAs. A, Class distribution of circRNAs, including 75.55% exonic, 9.16% intronic, 0.09% intergentic, 0.37% antisense and 14.84% sense overlapping circRNAs. B, Novel circRNAs and known circRNAs identified in the present study. C, Length distribution of circRNAs, which were mainly ranged from 80 to 2500 bp in length. D, Chromosomal distribution of circRNAs. Bp, base pair; Chr, chromosome; ChrM, mitochondrial genome

RNA sequencing detected a total of 9585 circRNAs in the control and DCM groups. The circRNAs were classified into five categories based on the locations in their host genes and included 7241 (75.55%) exonic, 878 (9.16%) intronic, 9 (0.09%) intergenic, 35 (0.37%) antisense and 1422 (14.84%) sense overlapping, (Figure 1A). A total of 6144 (64.1%) circRNAs had already been identified in previous studies and were included in the circBase database, while 3441 (35.9%) circRNAs were reported for the first time in the present study (Figure 1B). The majority of the circRNAs in the two groups ranged in length from 80 to 2500 bp (Figure 1C). The 9585 circRNAs were distributed across all chromosomes (Figure 1D).

### 3.2 Identification of differentially expressed circRNAs

**FIGURE 2:**
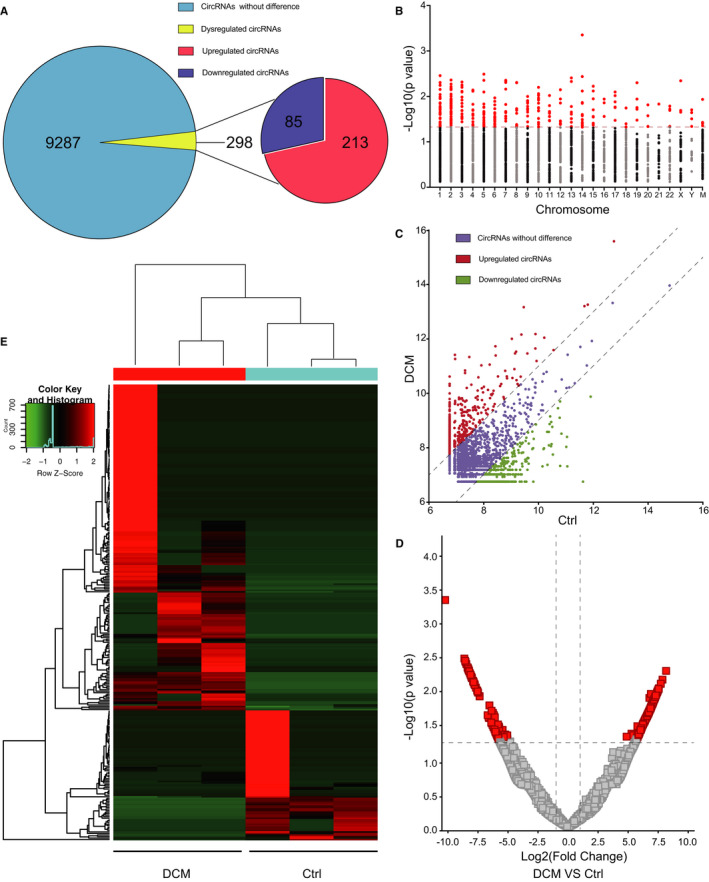
Identification of differentially expressed circRNAs. A, A total of 9585 circRNAs were detected by RNA sequencing. Among these, 298 circRNAs were differentially expressed between the control and DCM groups, including 213 up‐regulated and 85 down‐regulated circRNAs. B, A Manhattan plot of circRNAs with fold change ≥2 or ≤0.5 throughout all chromosomes. Red dots represent differentially expressed circRNAs. C, A scatter plot of the total circRNAs. D, A volcano plot of the total circRNAs. Red squares represent differentially expressed circRNAs. E, A heat map of dysregulated circRNAs. The criteria were a *P* value of <.05 and a fold change of ≥2 or ≤0.5. Ctrl, control; DCM, dilated cardiomyopathy

To assess the differentially expressed circRNAs, the criteria were set as a fold change of ≥2 or ≤0.5 and a *P* value of <.05. Compared with circRNAs in the control group, 298 dysregulated circRNAs were identified in patients with DCM, of which 231 were up‐regulated and 85 were down‐regulated (Figure 2A). A Manhattan plot presented the circRNAs with fold change ≥2 or ≤0.5 between two groups. We found that differentially expressed circRNAs (with a *P* value of <.05) were scattered throughout all chromosomes (Figure 2B). In addition, a scatter plot and a volcano plot were generated to identify differentially expressed circRNAs between the control and DCM groups (Figure 2C,D). A heat map of differentially expressed circRNAs is presented in Figure 2E.

### 3.3 Validation of circRNA expression

**FIGURE 3:**
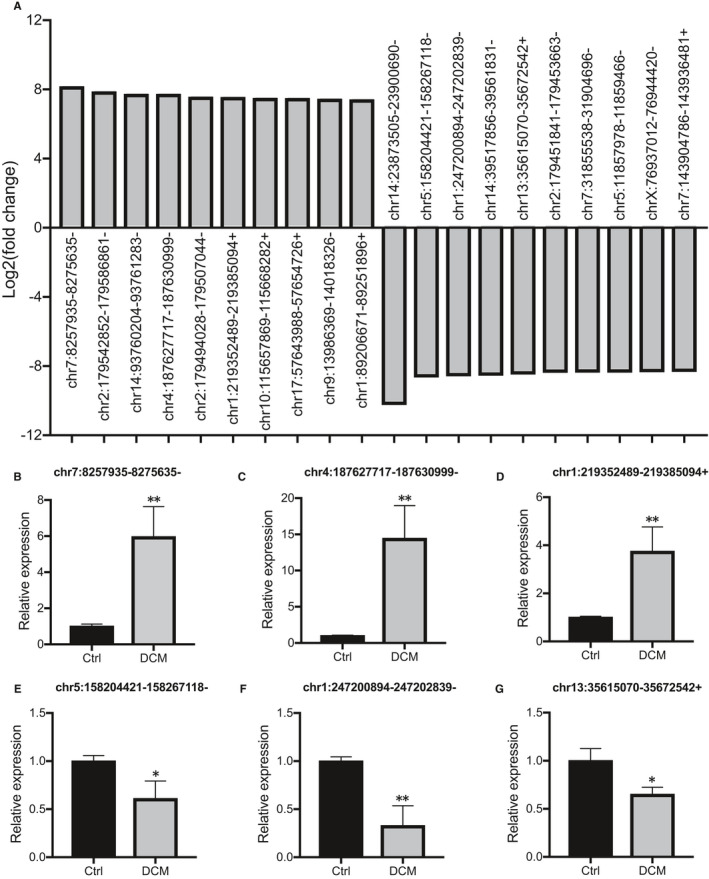
Validation of circRNA expression. A, Top 10 up‐regulated and top 10 down‐regulated circRNAs between the control and DCM groups. B‐G, Quantitative real‐time PCR was used to validate three up‐regulated and three down‐regulated circRNAs between the control (n = 3) and DCM (n = 3) groups. Three up‐regulated circRNAs: Chr7:8257935−8275635− (B), chr4:187627717−187630999− (C) and chr1:219352489−219385095+ (D). Three down−regulated circRNAs: Chr5:158204421−158267118− (E), chr1:247200894−247202839− (F) and chr13:35615070−35672542+ (G). The experiments were repeated for three times. ^*^ *P* < .05, ^**^ *P* < .01; Ctrl, control; DCM, dilated cardiomyopathy

To verify the expression levels of differentially expressed circRNAs, three up‐regulated and three down‐regulated circRNAs were selected from the top 10 up‐regulated circRNAs and top 10 down‐regulated circRNAs, respectively (Figure 3A). Details on the top 10 up‐regulated circRNAs and top 10 down‐regulated circRNAs are presented in Appendix S4. Chr7:8257935−8275635− (Figure 3B), chr4:187627717−187630999− (Figure 3C) and chr1:219352489−219385095+ (Figure 3D) were the three up‐regulated circRNAs. Chr5:158204421−158267118− (Figure 3E), chr1:247200894−247202839− (Figure 3F) and chr13:35615070−35672542+ (Figure 3G) were the three down‐regulated circRNAs. The qRT‐PCR results were consistent with the sequencing data.

### 3.4 Target miRNAs of differentially expressed circRNAs

**FIGURE 4:**
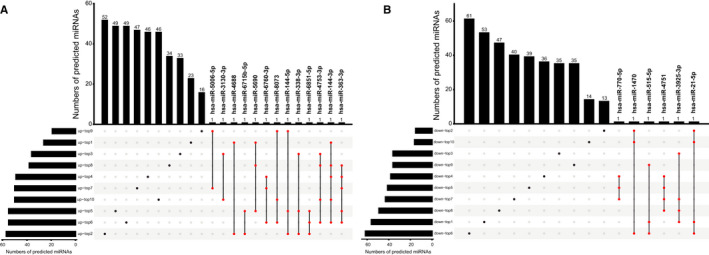
UpSet plots for target miRNAs of the circRNAs. Like Venn diagrams, UpSet plots are used to visualize set overlaps. The column at the bottom left shows the total number of target miRNAs of each circRNA. The black dot at the bottom right and the corresponding column above represent the number of target miRNAs that are not overlapped by other sets. The red dots with line and the corresponding column above represent the number of overlapping target miRNAs of sets. A, Target miRNAs and overlapping target miRNAs of the top 10 up‐regulated circRNAs. B, Target miRNAs and overlapping target miRNAs of the top 10 down‐regulated circRNAs. Up‐top1 to up‐top10, sets of target miRNAs of top 10 up‐regulated circRNAs; Down‐top1 to down‐top10, sets of target miRNAs of top 10 down‐regulated circRNAs

Next, we predicted the target miRNAs of differentially expressed circRNAs (top 10 up‐regulated and top 10 down‐regulated circRNAs). All the predicted miRNAs are presented in Appendix S5. We selected 13 miRNAs (hsa‐miR‐144‐5p, hsa‐miR‐338‐3p, hsa‐miR‐6715b‐5p, hsa‐miR‐3130‐3p, hsa‐miR‐5006‐5p, hsa‐miR‐8073, hsa‐miR‐5690, hsa‐miR‐4688, hsa‐miR‐6760‐3p, hsa‐miR‐6851‐5p, hsa‐miR‐4753‐3p, hsa‐miR‐144‐3p and hsa‐miR‐363‐3p), that were predicted to be targeted by at least two of the top 10 up‐regulated circRNAs (Figure 4A) and 6 miRNAs (hsa‐miR‐770‐5p, hsa‐miR‐4751, hsa‐miR‐1470, hsa‐miR‐515‐5p, hsa‐miR‐3925‐3p and hsa‐miR‐21‐5p) that were predicted to be targeted by at least two of the top 10 down‐regulated circRNAs (Figure 4B). The 19 miRNAs were used for the subsequent prediction of target mRNAs and construction of ceRNA networks.

### 3.5 Identification of differentially expressed mRNAs and validation of mRNA expression

**FIGURE 5:**
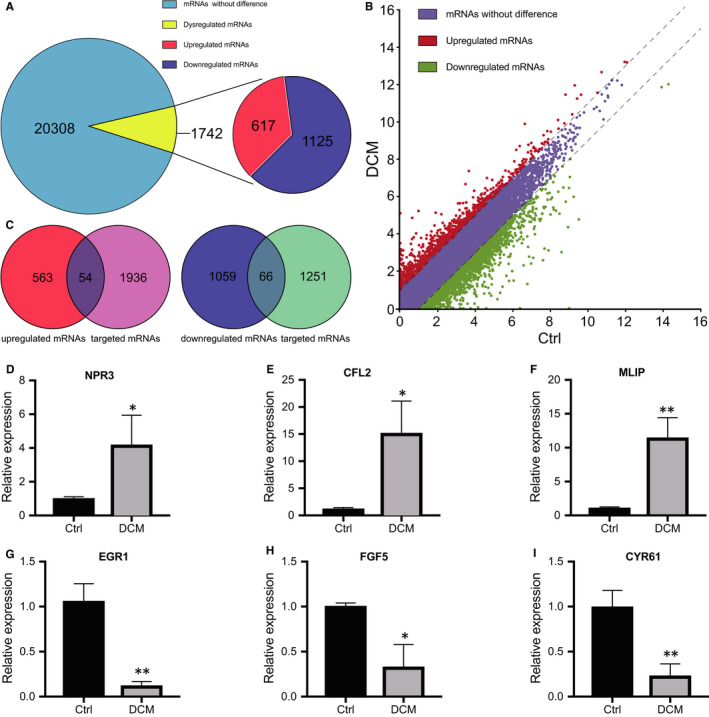
Identification of differentially expressed mRNAs and validation of mRNA expression. A, A total of 22050 mRNAs were detected by RNA sequencing. Among these, 1742 mRNAs were differentially expressed between the control and DCM groups, including 617 up‐regulated and 1125 down‐regulated mRNAs. B, A scatter plot of the total mRNAs. C, Overlapping number of up‐regulated mRNAs and target mRNAs of the top 10 up‐regulated circRNAs. Overlapping number of down‐regulated mRNAs and target mRNAs of the top 10 down‐regulated circRNAs. D‐I, Quantitative real‐time PCR was used to validate three up‐regulated and three down‐regulated mRNAs between the control (n = 3) and DCM (n = 3) groups. Three up‐regulated mRNAs: NPR3 (D), CFL2 (E) and MLIP (F). Three down‐regulated mRNAs: EGR1 (G), FGF5 (H) and CYR61 (I). The experiments were repeated for three times. ^*^ *P* < .05, ^**^ *P* < .01; Ctrl, control; DCM, dilated cardiomyopathy

RNA sequencing detected a total of 22050 mRNAs in the control and DCM groups. According to the criteria of a *P* value of <.05 and a fold change of ≥2 or ≤0.5, 1742 mRNAs were differentially expressed in the DCM group compared with the control group, of which 617 were up‐regulated and 1125 were down‐regulated (Figure 5A). A scatter plot was generated to identify differentially expressed circRNAs between the control and DCM groups (Figure 5B). Next, we predicted the target mRNAs of the selected miRNAs shown in Figure 4A,B and compared the predictions with our mRNA sequencing data. Fifty‐four target mRNAs of the 13 selected miRNAs were up‐regulated, and 66 target mRNAs of the 6 selected miRNAs were down‐regulated (Figure 5C). We selected three mRNAs from the 54 up‐regulated mRNAs and three mRNAs from the 66 down‐regulated mRNAs to verify their expression levels. NPR3 (Figure 5D), CFL2 (Figure 5E) and MLIP (Figure 5F) were the three up‐regulated mRNAs. EGR1 (Figure 5G), FGF5 (Figure 5H) and CYR61 (Figure 5I) were the three down‐regulated mRNAs. The qRT‐PCR results were consistent with the sequencing data.

### 3.6 GO and KEGG pathway analyses of miRNA target mRNAs

**FIGURE 6:**
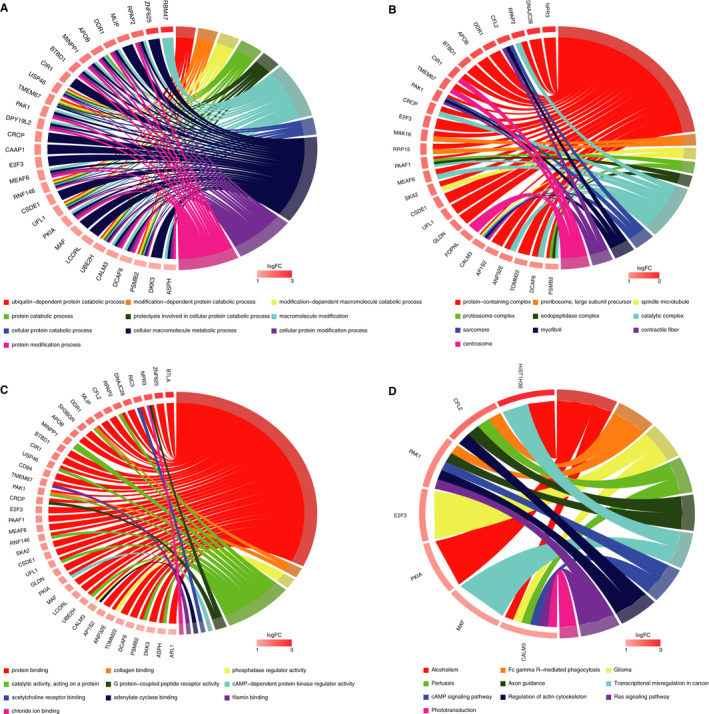
GO and KEGG pathway analyses of 54 up‐regulated target mRNAs. Chord plots indicate enrichment analysis of these mRNAs. A, Biological process of GO analysis. B, Cellular component of GO analysis. C, Molecular function of GO analysis. D, KEGG pathway analysis. GO, Gene Ontology; KEGG, Kyoto Encyclopedia of Genes and Genomes

**FIGURE 7:**
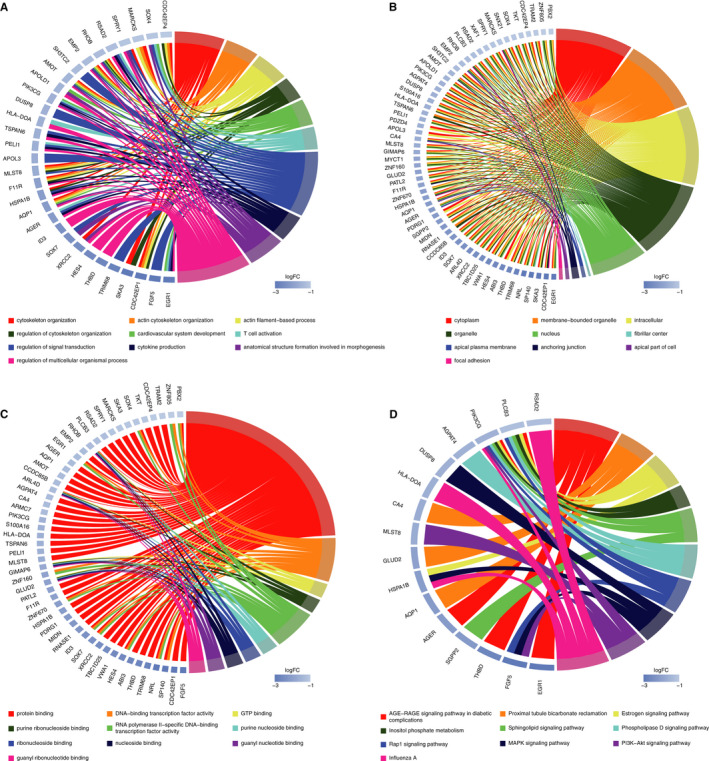
GO and KEGG pathway analyses of 66 down‐regulated target mRNAs. Chord plots indicate enrichment analysis of these mRNAs. A, Biological process of GO analysis. B, Cellular component of GO analysis. C, Molecular function of GO analysis. D, KEGG pathway analysis. GO, Gene Ontology; KEGG, Kyoto Encyclopedia of Genes and Genomes

To understand the potential functions of the top 10 up‐regulated and top 10 down‐regulated circRNAs, GO and KEGG pathway analyses were performed based on the 54 up‐regulated mRNAs and 66 down‐regulated mRNAs, respectively (details on the mRNAs are presented in Appendix S6). The up‐regulated mRNAs were found to be mostly enriched in the following terms: cellular macromolecule metabolic process and cellular protein modification in the ‘biological process’ analysis (Figure 6A); protein‐containing complex in the ‘cellular component’ analysis (Figure 6B); and protein binding in the ‘molecular function’ analysis (Figure 6C). The down‐regulated mRNAs were found to be mostly enriched in the following terms: regulation of multicellular organismal process and regulation of signal transduction in the ‘biological process’ analysis (Figure 7A); cytoplasm, nucleus and organelle in the ‘cellular component’ analysis (Figure 7B); and protein binding in the ‘molecular function’ analysis (Figure 7C). KEGG pathway analysis revealed that the up‐regulated mRNAs were mainly associated with Ras signalling pathway, regulation of actin cytoskeleton and cAMP signalling pathway (Figure 6D), and the down‐regulated mRNAs were mainly associated with MAPK signalling pathway, phospholipase D signalling pathway and Rap1 signalling pathway (Figure 7D).

### 3.7 Prediction of ceRNA networks

**FIGURE 8:**
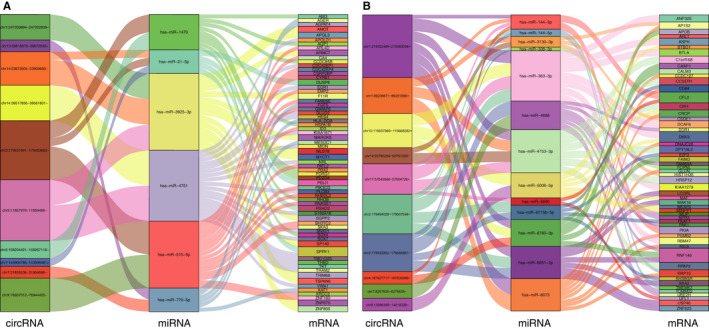
Sankey diagrams for the ceRNA networks in human DCM. Each rectangle represents a circRNA/miRNA/mRNA, and the connection degree is visualized based on the size of the rectangle. A, The ceRNA network of the top 10 down‐regulated circRNAs. B, The ceRNA network of the top 10 up‐regulated circRNAs

According to the ceRNA theory, circRNAs and mRNAs that share the same miRNA‐binding sites can function as ceRNAs and should be both either up‐regulated or down‐regulated, so we constructed two ceRNA networks using dysregulated circRNAs and mRNAs. One network included the top 10 down‐regulated circRNAs, 6 predicted miRNAs of the top 10 down‐regulated circRNAs and 66 down‐regulated mRNAs (Figure 8A). The other network comprised the top 10 up‐regulated circRNAs, 13 predicted miRNAs of the top ten up‐regulated circRNAs and 54 up‐regulated mRNAs (Figure 8B). The ceRNA networks are complicated, and circRNAs can indirectly interact with mRNAs by directly binding to miRNAs. As shown in Figure 8A, a down‐regulated circRNA was less effective at absorbing target miRNAs, resulting in the down‐regulation of miRNA‐targeted mRNAs. As shown in Figure 8B, an up‐regulated circRNA could more effectively absorb target miRNAs, resulting in the up‐regulation of miRNA‐targeted mRNAs. Therefore, when circRNAs and mRNAs act as ceRNAs, both are up‐regulated or down‐regulated.

## 4 DISCUSSION

DCM, characterized by systolic dysfunction and ventricular dilation, is a major cause of heart failure and cardiac transplantation worldwide. Due to the severe outcome of DCM, it is imperative to find related effective diagnostic biomarkers and therapeutic targets. Up to data, DCM has been considered a genetic disease, and more than 30 genes have been reported to be related to DCM. However, recent studies found that gene mutations alone might not fully explain the genesis and development of DCM.^26^ Over the past few years, many researchers have made great efforts to explore the molecular mechanisms of DCM, and ncRNAs have been found to play important roles in the development of DCM.^23^, ^27^ Although circRNAs are extensively studied in animal disease models, their functions in humans remain to be elucidated.

In the present study, we performed an analysis of dysregulated cardiac circRNAs and mRNAs between patients with DCM and healthy controls and constructed two circRNA‐miRNA‐mRNA networks. A total of 9585 circRNAs were identified in the DCM and control groups, of which 231 circRNAs were up‐regulated and 85 circRNAs were down‐regulated. In addition, we obtained 22050 mRNAs in the two groups, of which 617 were significantly up‐regulated and 1125 were significantly down‐regulated. The top 10 up‐regulated and top 10 down‐regulated circRNAs were selected for predictions of their interacting miRNAs and circRNA‐targeted mRNAs. In the circRNA‐miRNA‐mRNA network, circRNAs and mRNAs acts as ceRNAs, and according to the ceRNA theory, the circRNAs and mRNAs should be both either up‐regulated or down‐regulated and share the same miRNA‐binding sites. We compared the mRNA sequencing results and the predicted results and identified 54 up‐regulated circRNA‐targeted mRNAs and 66 down‐regulated circRNA‐targeted mRNAs.

To evaluate the potential functions of the dysregulated circRNAs in the ceRNA networks, GO and KEGG signalling pathway analyses were performed using the circRNA‐targeted mRNAs. We found that the down‐regulated mRNAs were mainly enriched in actin cytoskeleton organization, actin filament‐based process and cardiovascular system development in the ‘biological process’ analysis, which means that these processes were down‐regulated and the cardiac systolic function would probably be inhibited. The up‐regulated and down‐regulated mRNAs were both mainly enriched in protein binding in ‘molecular function’ analysis. KEGG pathway analysis revealed that down‐regulated mRNAs were also involved in the oestrogen signalling pathway, PI3K signalling pathway and inositol phosphate metabolism. It has been reported that oestrogen can attenuate left ventricular and cardiomyocyte hypertrophy in an oestrogen receptor‐dependent pathway that increases calcineurin degradation.^28^ In addition, the activation of the oestrogen signalling pathway could attenuate chronic volume overload‐induced structural and functional remodelling in male rat hearts.^29^ Inositol phosphate metabolism has been reported to be associated various physiological and pathophysiological processes. In the cardiomyocytes, the metabolite of inositol phosphate could induce the release of Ca^2+^ in the cytoplasm and increase the concentration of Ca^2+^, which provides energy for the contraction of cardiomyocytes. Inositol phosphate metabolism also has effects on actin remodelling and changes in the cytoskeleton.^30^ PI3K signalling pathway is an important part of intracellular signal transduction. Recent studies have placed PI3K signalling pathway at the centre of the regulation of key homeostatic processes in the cardiovascular system, which are directly or indirectly linked to modulation of Ca^2+^ fluxes and are associated with the development of various cardiovascular diseases, including heart failure.^31^ The results of GO and KEGG signalling pathway analyses indicated that the circRNAs in the ceRNA networks were mainly associated with cardiac systolic and diastolic function, a lack of which would lead to DCM.

Recent studies have revealed that circRNAs play important roles in cardiac development and are involved in the pathophysiological processes of cardiovascular diseases.^32^ A study reported that circRNAs derived from the TTN gene were differentially expressed in neonatal and adult rat hearts, indicating a critical role for circRNAs in heart growth.^33^ Similarly, circRNAs were found to be differentially expressed during the differentiation of cardiac progenitors into cardiomyocytes, suggesting that circRNAs may participate in the cardiac cell specification.^34^ In addition to their functions in cardiac development, circRNAs also play critical roles in cardiac dysfunction. For instance, heart‐related circRNA (HRCR) was reported to act as an endogenous miRNA‐223 sponge to inhibit the activity of miR‐233 and thus inhibit cardiac hypertrophy and heart failure.^35^ CircRNA CDR1as was reported to increase cardiac infarct size by regulating the expression of miR‐7 target genes.^36^ CircRNAs were also found to be associated with cardiac fibrosis, atherosclerosis, arrhythmia and so on.^21^ In addition, compared with the functions of miRNAs and lncRNAs, the functions of circRNAs in cardiac development and cardiovascular diseases are still less understood. The ceRNA networks constructed in the present study, together with the GO and KEGG analyses, indicated that circRNAs could play critical roles in various biological processes. These results broadened our understanding of the mechanism of cardiovascular diseases.

In conclusion, our study evaluated cardiac circRNA expression in DCM by RNA sequencing and identified the potential functions of dysregulated circRNAs using bioinformatics. More importantly, we constructed two circRNA‐miRNA‐mRNA networks based on the ceRNA theory. In the networks, one circRNA can indirectly affect multiple mRNAs by binding to multiple miRNAs, indicating that circRNAs might be involved in a complex mechanism in the development of DCM. These findings provide novel insight into the pathogenesis of DCM and a theoretical basis for future studies of circRNAs in DCM.

## CONFLICT OF INTERESTS

The authors declare that there are no conflicts of interest.

## AUTHOR CONTRIBUTIONS

**Zhenhao Lin:** Conceptualization (equal); Data curation (equal); Validation (equal); Visualization (lead); Writing‐original draft (lead). **Yongchao Zhao:** Conceptualization (equal); Data curation (equal); Investigation (equal); Methodology (equal). **Fangjie Dai:** Investigation (equal); Methodology (equal); Validation (equal). **Enyong Su:** Investigation (equal); Methodology (equal); Validation (equal). **Fuhai Li:** Investigation (equal); Methodology (equal). **Yan Yan:** Funding acquisition (lead); Writing‐review & editing (supporting).

## Supporting information

Appendix S1

Appendix S2

Appendix S3

Appendix S4

Appendix S5

Appendix S6

## Data Availability

All sequence data discussed in this study are accessible through GEO Series accession number GSE162505.
